# Genetic Insights into *Graminella nigrifrons* Competence for *Maize fine streak virus* Infection and Transmission

**DOI:** 10.1371/journal.pone.0113529

**Published:** 2014-11-24

**Authors:** Bryan J. Cassone, Fiorella M. Cisneros Carter, Andrew P. Michel, Lucy R. Stewart, Margaret G. Redinbaugh

**Affiliations:** 1 United States Department of Agriculture- Agricultural Research Service, Corn, Soybean and Wheat Quality Research Unit, Ohio Agricultural Research and Development Center (OARDC), Wooster, Ohio, United States of America; 2 Department of Plant Pathology, Ohio Agricultural Research and Development Center (OARDC), The Ohio State University, Wooster, Ohio, United States of America; 3 Department of Entomology, The Ohio State University, Ohio Agricultural Research and Development Center (OARDC), The Ohio State University, Wooster, Ohio, United States of America; United States Department of Agriculture, Beltsville Agricultural Research Center, United States of America

## Abstract

**Background:**

Most plant-infecting rhabdoviruses are transmitted by one or a few closely related insect species. Additionally, intraspecific differences in transmission efficacy often exist among races/biotypes within vector species and among strains within a virus species. The black-faced leafhopper, *Graminella nigrifrons*, is the only known vector of the persistent propagative rhabdovirus *Maize fine streak virus* (MFSV). Only a small percentage of leafhoppers are capable of transmitting the virus, although the mechanisms underlying vector competence are not well understood.

**Methodology:**

RNA-Seq was carried out to explore transcript expression changes and sequence variation in *G. nigrifrons* and MFSV that may be associated with the ability of the vector to acquire and transmit the virus. RT-qPCR assays were used to validate differential transcript accumulation.

**Results/Significance:**

Feeding on MFSV-infected maize elicited a considerable transcriptional response in *G. nigrifrons*, with increased expression of cytoskeleton organization and immunity transcripts in infected leafhoppers. Differences between leafhoppers capable of transmitting MFSV, relative to non-transmitting but infected leafhoppers were more limited, which may reflect difficulties discerning between the two groups and/or the likelihood that the transmitter phenotype results from one or a few genetic differences. The ability of infected leafhoppers to transmit MFSV did not appear associated with virus transcript accumulation in the infected leafhoppers or sequence polymorphisms in the viral genome. However, the non-structural MFSV 3 gene was expressed at unexpectedly high levels in infected leafhoppers, suggesting it plays an active role in the infection of the insect host. The results of this study begin to define the functional roles of specific *G. nigrifrons* and MFSV genes in the viral transmission process.

## Introduction

Plant-infecting rhabdoviruses require arthropod vectors for their transmission to new host plants. There are more than 90 known plant rhabdoviruses, most of which are transmitted by hemipteran insects, including aphids (Aphididae), planthoppers (Delphacidae), and leafhoppers (Cicadellidae) [1]. Plant rhabdoviruses are transmitted in a persistent propagative manner, replicating in and migrating through the insect across several molecular and physical barriers prior to being transmitted to a new plant host [1]. Following virus acquisition and before the vector becomes inoculative, there is a latency period of a few days to weeks, during which the virus replicates in the vector [1]–[4]. The movement and replication of rhabdoviruses in their insect hosts requires specific interactions between virus and vector components to overcome the major transmission barriers, which usually results in a high degree of vector specificity.

Differences in rhabdovirus transmission efficiency have been reported among races/biotypes within the same vector species, and also among virus strains transmitted by the same insect vector [2], [5], [6]. *Graminella nigrifrons* is a common leafhopper on wheat, maize, and other grasses in the eastern U.S [7], [8]. It is the only known vector of *Maize fine streak virus* (MFSV; family *Rhabdoviridae*, genus *Nucleorhabdovirus*) [9], [10]. However, within a laboratory population, only a small percentage of *G. nigrifrons* exposed to MFSV were capable of transmitting the virus [10]. In the remainder of the population, some individuals were hosts of but did not transmit MFSV, and others were not virus hosts. These groups of *G. nigrifrons* were categorized as ‘transmitters’, which acquired and transmitted virus (i.e., they are vectors); ‘acquirers’, which acquired but did not transmit MFSV; and, ‘non-acquirers’, which did not retain virus after feeding on maize infected with MFSV [11]. The underlying mechanisms that determine the transmission efficacy of MFSV and other rhabdoviruses are not well understood.

Several intrinsic and extrinsic factors are thought to influence the capability of insects to acquire, maintain, and transmit pathogens [3], [12]. However, traits that have a genetic component are of the greatest significance—a discordant genetic interaction will not result in competent vectors even if the environment is suitable [13], [14]. It is thought that genetic elements in both the vector and virus and their interactions determine whether an individual insect within a species is capable of vectoring a particular virus strain [15]. Assessment of pathogen-induced transcriptional changes in insect vectors has been key for identifying genes contributing to pathogen host responses, and providing insights into the molecular and cellular processes important for pathogen infection and transmission [16].

Currently, investigations into the regulatory control of insect immune systems are most advanced for *Drosophila* and *Anopheles* but data are becoming increasingly available for plant-infecting vectors [17], [18]. Our recent work indicated *G. nigrifrons* responds to feeding on MFSV-infected plants by increasing transcript levels of genes involved in immune defenses [19]. However, in these experiments, transcript abundance was characterized without regard to the transmission competence of individual leafhoppers. While leafhopper populations composed only of transmitters, acquirers, or non-acquirers have yet to be selected, a combination of laboratory and greenhouse assays has been developed to distinguish individuals based on their transmission group [11]. We hypothesized that the differences in MFSV transmission capability among individual leafhoppers have a significant genetic component (e.g., regulatory or sequence divergence).

The sequencing and assembly of the MFSV genome [20] and *G. nigrifrons* transcriptome [11] provide valuable tools for exploring the transcriptional basis of vector competence. We hypothesized that transcript expression and sequence variation in the vector and virus contribute to MFSV infection of and transmission by *G. nigrifrons*, and used RNA-Seq and RT-qPCR approaches to examine differences among *G. nigrifrons* transmission groups and MFSV. This study provides novel insights into the genetic mechanisms underlying the capacity of *G. nigrifrons* to transmit MFSV.

## Methods

### Leafhopper colony and virus maintenance

Experiments were carried out using a laboratory colony of *G. nigrifrons* established from multiple, ongoing field collections taken near Wooster, OH since the early 1980s (40.8050°N, 81.9353°W). Leafhoppers were maintained on maize (*Zea mays* L. ‘Early Sunglow’, Schlessman Seed Co., Milan, OH) as previously described [21], [22]. Growth chamber conditions for all experiments consisted of 28°C/16 h day and a 22°C/8 h night cycle.

The MFSV isolate was originally isolated from southwestern Georgia (U.S.A.) [9] and was maintained in the sweetcorn hybrid ‘Early Sunglow’ by serial transmission with *G. nigrifrons* [10]. To produce MFSV-infected experimental plants, symptomatic leaf tissue was ground in 0.1 M potassium phosphate, pH 7.0 (1∶4 g/ml) and inoculated onto healthy seeds of the sweetcorn hybrid ‘Spirit’ by vascular puncture inoculation (VPI) as previously described [9]. At two days post-inoculation (dpi), seeds were planted into greenhouse soil and moved a growth chamber.

### Insect rearing and MFSV transmission assays

For virus acquisition, gravid *G. nigrifrons* females were placed on symptomatic MFSV-infected maize for oviposition. After 2 d, adults were removed and nymphs were allowed to feed on the infected plants for 6 wks. Fresh symptomatic plants were added every 2 wks during the acquisition period. Subsequently, 300 adults were randomly selected and individually transferred to healthy ‘test’ plants (4 d old ‘Spirit’ seedlings) for a 7 d inoculation period. After the 7 d, leafhoppers were harvested and individually stored in RNase-free tubes at −80°C until RNA isolation. Test plants were then moved to a greenhouse for 4 wks for symptom development.

For the experimental treatments, *G. nigrifrons* individuals were separated into transmitters, acquirers, and non-acquirers as previously described [11]. Briefly, an insect was designated as a transmitter if the corresponding test plant developed symptoms and MFSV was detected in the leafhopper by RT-PCR, as outlined below. Individuals were designated acquirers if MFSV was detected by RT-PCR, but the test plant did not develop symptoms, and non-acquirers if they were negative by both assays (test plant symptom development and RT-PCR). *G. nigrifrons* fed on healthy ‘Spirit’ maize for the same time period and reared under identical conditions as the treatment leafhoppers were collected as the healthy control. The experiment (treatments and control) was replicated three times using leafhoppers from different cohorts.

### RNA isolation

Total RNA was isolated from *G. nigrifrons* using Trizol (Life Technologies, Carlsbad, CA) following the manufacturer's protocol. DNA was removed before RNA quantification with Turbo DNAfree (Ambion, Inc, Austin TX). DNA-free RNA was quantified and quality evaluated using the Experion Automated Electrophoresis System (Bio-Rad Laboratories Inc. Hercules, CA). Only those RNAs with RNA Quality (RQ) numbers of 7 or higher were used in further analyses.

### RT-PCR assays

To identify transmitters, acquirers, and non-acquirers, one-step RT-PCR assays (Promega AccessQuick RT-PCR system, Madison, WI) were carried using 100 ng of DNA-free total RNA (see above) isolated from individual *G. nigrifrons*. The primer pair 514F (5′-GTGCAGAATTGCCCTATCC-3′)/1631R (5′-TCGAGGCAATTCCTGTATC -3′) was used to amplify a 1117 nt fragment corresponding to the MFSV N gene (GenBank accession number NC_005974). Reverse transcription was carried out at 45°C for 45 min. PCR included an initial denaturation at 94°C for 3 min, 35 cycles of 94°C for 30 sec (denaturation), 55°C for 30 sec (annealing) and 72°C for 1 min (extension) followed by a final extension step at 72°C for 10 min. Total RNA isolated from MFSV-infected plant leaves was used a positive control and RNase-free water served as the negative control. Amplicons were visualized on 1% agarose gels.

### cDNA library preparation

Adaptor tagged ds-cDNA libraries were constructed from total DNA free-RNA (1 µg per sample) using the TruSeq Sample Prep Kit V1 and V2 (Illumina, San Diego, CA). Each sample consisted of 10 pooled leafhoppers of the same cohort and transmission group. The quality of the cDNA libraries was assessed using the Experion Automated Electrophoresis System and quantified using the Qubit 2.0 Fluorometer (Life Technologies, Carlsbad, CA). Samples were diluted to 18 nM and pooled to generate the multiplexed cDNA library. In total, 12 adaptor-tagged samples were pooled, consisting of a healthy control and three treatments (transmitters, acquirers and non-acquirers) for each of the three replicate experiments.

### Next generation sequencing and raw read preprocessing

The cDNA library (50 fmoles) was sequenced on one flow cell lane using the Illumina HiSeq2000 at the Ohio State University Comprehensive Cancer Center. Four fluorescently-labeled nucleotides and a specialized polymerase were used to determine the cluster sequence base by base in parallel. The mean library insert sequence size was 269 bp and both ends of the library were sequenced to generate 100 bp paired-end reads. The Illumina Analysis Package CASAVA 1.8.2 was used to perform bcl (base calls per cycle) conversion and demultiplexing. Image deconvolution and quality value calculations were carried out using the Illumina GA pipeline v1.6.

Raw reads were imported into CLC Genomics Workbench (v6.0.1, CLC Bio) and trimmed for quality, adapter indexes, and poly(A) tails using the default settings (Ambiguous limit = 2, quality limit = 0.05). Redundant reads were removed using the Duplicate Removal plugin in CLC. The raw sequence reads can be retrieved from the NCBI short sequence read archive under the accession number SRP032742. Preprocessed reads were mapped to the *G. nigrifrons* reference transcriptome assembly [19] using the map to reference function in CLC Genomics Workbench v6.0.1 and default settings. The subset of expressed transcripts was determined from the mapped read data files as containing a minimum four mapped reads for any two replicates in at least one treatment.

### Transcript expression analysis

Using custom R scripts, *de novo* assembled transcript read counts (paired only) were normalized by calculating the number of unique reads (i.e. mapping to only one transcript in the *G. nigrifrons* transcriptome) per kilobase of exon model per million mapped reads (RPKM) [23]. Quality assessment of the normalized data file was conducted using the quality control function of transcriptome analysis in CLC Bio.

Mapped read count files were imported into Bioconductor [24] an open-source software project based on the R programming language. Bayes-moderated independent sample *t*-tests were carried out using the limma package in R to identify the sets of transcripts differentially expressed between the control group and each transmission group separately (three contrasts in total). Expression was considered to be significantly different at *P*<0.05 (FDR<0.20). For comparison of statistical methods, differentially expressed transcripts were also identified independently using the R module edgeR [25], which uses an overdispersed Poisson model to moderate the dispersion. One-way analysis of variance (ANOVA) between treatments was conducted on the subset of transcripts that were differentially expressed in one or more contrasts using the nlme package in R (*P*<0.05). The set of differentially expressed transcripts were filtered to include only the subset with a mean RPKM >2 between treatments.

Functional annotation of transcripts was carried out using desktop downloaded BLASTx software against the nr database (*E*-value<10^−6^). The DAVID v6.7 annotation clustering module [26], [27] was used to classify differentially expressed transcripts into functional groups. Transcripts were first converted to *D. melanogaster* transcript IDs, then enrichment of GO and other annotation terms in candidate sublists were explored using the functional annotation clustering tool. The enrichment score ranks the biological significance of gene groups based on overall EASE scores (modified Fisher's Exact Test) of all enriched annotation terms, thereby accounting for the relative importance of the groups as part of an exploratory rather than strictly statistical analysis. Significant clusters were defined using the default parameters.

### Insect vector and virus sequence polymorphism analysis

Single nucleotide polymorphisms (SNPs), insertions, and deletions were identified among treatments in *G. nigrifrons* transcripts and between MFSV transcripts from transmitters and acquirers using the quality variant detection re-sequencing function (based on the Neighborhood Quality Standard (NQS) algorithm) in the CLC Genomics Workbench. Detection of vector and virus polymorphisms was based on the *G. nigrifrons* reference transcriptome [19] and MFSV genome [20], respectively. Only homozygous polymorphisms fixed between treatments in all replicates with coverage of ≥8 paired-end reads were considered. Subsequently, SNPs that putatively result in non-synonymous differences in vector and virus proteins were identified. Open reading frame (ORF) polypeptide sequences were predicted from the transcript sequences using ORFPredictor [28]. ClustalW v2.0 [29] was used to align polypeptide sequences for the homologous ORF from each treatment to detect potential amino acid changes.

### RT-qPCR assays

Differential accumulation of selected transcripts was validated between the *G. nigrifrons* transmitters and healthy control using a two-step quantitative RT-PCR and three samples per treatment. Samples consisted of 1 µg of DNA-free total RNA pooled from 10 leafhoppers corresponding to the same transmission group. cDNA was generated for each sample using ThermoScript Reverse Transcriptase (Life Technologies, Carlsband, CA) following the manufacturer's protocol. The annealing and elongation steps were performed at 65°C for 5 min and at 60°C for 60 min, respectively. The reactions were terminated at 85°C for 5 min, and cDNA was stored at −20 C. No reverse transcriptase was added to the negative control (NRT). Primer pairs for targeted mRNAs were designed using Primer3 [30] and are shown in Table S1. Primer efficiency (*E*) was evaluated by performing a dilution series experiment using each primer pair and the equation *E* = 10^(−1/slope)^ [31]. The *ribosomal protein* S13 (RPS13) was used as the endogenous qPCR control gene [11].

The qPCR reactions (15 µl) reactions containing 1X SsoFast EvaGreen Supermix (Bio-Rad Laboratories Inc., Hercules, CA), 300 nM of each primer and 1 µl of a 10-fold dilution of cDNA were incubated at 98°C for 2 min followed by 40 cycles of 98°C for 2 sec and 55°C for 5 sec. After the amplification, a melting curve protocol was performed between 70 and 90°C with increments of 0.2°C every 10 sec. No cDNA template was added to the negative control (NTC). All reactions were performed with two technical replicates using the Bio-Rad CFX96 Touch Real-Time PCR Detection System (Bio-Rad Laboratories, Inc.).

Transcript accumulation levels were measured separately for each reference and target gene. Relative transcript abundance was calculated using the 2^−ΔΔC^_T_ method [32]. Threshold cycle (*C*_T_) values reported by the CFX96 Real-Time PCR Detection System were normalized to the control gene and converted to relative log_2_-fold differences between treatments. One-tailed independent *t*-tests (*P*<0.05) were used to assess differential expression between treatments for both the vector and viral genes.

## Results and Discussion

In these experiments, we examined transcript expression and sequence changes in *G. nigrifrons* and MFSV that might be linked to insect vector competence. *G. nigrifrons* were fed on MFSV-infected maize for six weeks followed by one week on healthy plants, then evaluated for their ability to acquire and transmit MFSV. Twelve cDNA libraries were constructed and sequenced: one each for non-acquirers, acquirers, transmitters, and leafhoppers fed on healthy plants in each of three independent experiments. The sequence analysis generated ∼231 million raw 100 bp paired end reads. After preprocessing and duplicate read removal, 9.1 to 20.5 million non-redundant reads were obtained per sample. The reads were mapped to the *G. nigrifrons* reference transcriptome [19] and the MFSV genome [20]. Roughly 91% (n = 159,477,621) and <1% (n = 432,056) of reads mapped to *G. nigrifrons* and MFSV, respectively. A total of 34,732 *G. nigrifrons* transcripts were detected as expressed and all seven MFSV genes were expressed.

### Few *G. nigrifrons* individuals transmit MFSV

Among the three biological replicates, 900 leafhoppers were assayed for MFSV infection and transmission efficiency to test plants using symptom development and RT-PCR. One-quarter (24±5%) of leafhoppers were infected with MFSV and fewer (16.7±5.3%) were capable of transmitting the virus, consistent with previous findings [11]. Although transmission efficiencies were low in comparison to other insect vector – rhabdovirus systems [21], [33]–[36], the consistency among replicates and with previous studies using the same leafhopper colony suggests that the intraspecific variation in MFSV transmission capacity has a strong genetic component [21].

### Feeding on MFSV-infected maize elicits a transcriptional response in *G. nigrifrons*

Differences in transcript expression between leafhoppers in each transmission group and the control group fed on healthy maize were identified using Bayes-moderated *t*-tests (*P*<0.05). Across the three transmission groups, 14,062 differentially expressed transcripts were identified. Most of these transcripts (87%) were also identified using the edgeR package, indicating good correlation between the two statistical approaches. More differentially expressed transcripts were detected in transmitters (n = 10,417) and acquirers (n = 8,619) than in non-acquirers (n = 4,910). Thus, between 13% and 30% of *G. nigrifrons* transcripts were differentially expressed in the three treatments, suggesting that six weeks of feeding on MFSV-infected plants followed by one week on healthy plants impacted the transcriptional profiles of leafhoppers, regardless of whether they become infected with the virus or not. Of the differentially expressed transcripts, 2,695 were shared across the three transmission groups.

Differential accumulation of six transcripts was verified by RT-qPCR in *G. nigrifrons* (Table 1). The six transcripts were identified by RNA-Seq as up or downregulated in one transmission group relative to the other transmission groups and the healthy control. Similar significant differences in transcript expression were detected for all six transcripts using RNASeq and RT-qPCR, indicating transcript expression differences identified by RNASeq were valid.

**Table 1:** Comparison of abundance for six differentially expressed transcripts in *G. nigrifrons* transmitters or acquirers relative to the healthy control using RNA-Seq and RT-qPCR.

Transcript ID^a^	Transmission Group	Putative Function^b^	RNA-Seq^c^	RT-qPCR^d^
GnigEST-4730^e^	Transmitter	DNA2-like helicase-like isoform	2.53	2.62
GnigEST-9819	Transmitter	alanine–glyoxylate aminotransferase	3.27	3.37
GnigEST-37813	Transmitter	Unknown	3.12	3.59
GnigEST-6190	Transmitter	retinol dehydrogenase	−3.73	−4.01
GnigEST-10957	Acquirer	Unknown	3.91	3.69
GnigEST-19305	Acquirer	histone-lysine N-methyltransferase	3.84	3.88

a Transcript IDs were derived from *de novo* assembly (Table S2).

b Functional description were derived from orthologs identified by BLASTx against the nr database.

c Fold-change expression in transmitters relative to healthy control calculated from RNA-Seq RPKM.

d Fold-change expression in RT-qPCR in transmitters relative to healthy control calculated from 2^−ΔΔC^_T_.

e GnigEST represents the universal identifier for transcripts in the *G. nigrifrons* transcriptome, and each transcript has a unique number (between 1 and 58717). The sequences are deposited in the NCBI Transcriptome Shotgun Assembly archive under the accession number GAQX00000000.

### Differential transcript accumulation among *G. nigrifrons* transmission groups

**Figure 1:**
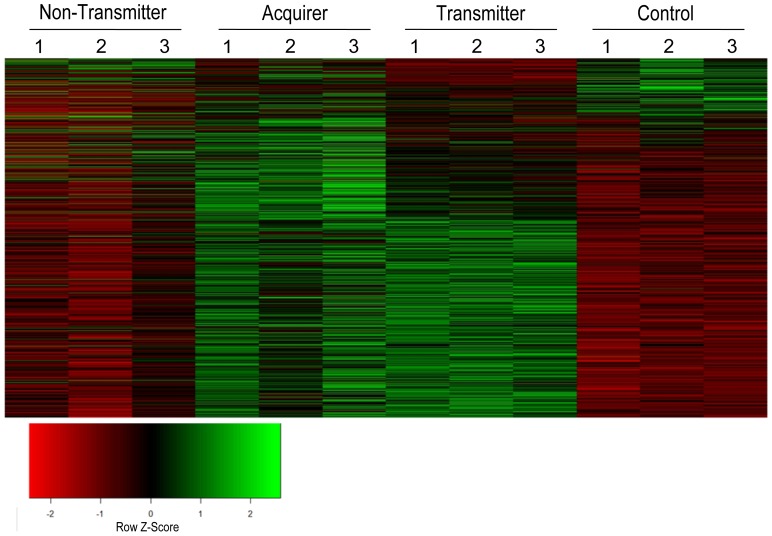
Heat map for expression of 891 differentially expressed *G. nigrifrons* transcripts. Differentially expressed transcripts were identified using one-way ANOVA (*P*<0.05; RPKM change >2). Each row represents an individual transcript; each column labeled 1–3 represent replicate samples of non-acquirers, acquirers, transmitters, or the healthy control, as outlined in the Methods. For each transcript (row), the relative expression level for each sample is represented by a color that reflects its z-score (shown in the redgreen key), calculated by subtracting the mean expression value for the row from the sample value and dividing by the standard deviation for the row.

Differences in transcript expression among the three transmission groups were examined in the subset of 14,062 transcripts that differentially accumulated in *G. nigrifrons* feeding on MFSV-infected maize using one-way ANOVA (*P*<0.05). A total of 891 (6%) transcripts accumulated differentially among groups (Table S2). *Drosophila* orthologs were identified for nearly 40% (n = 348) of the differentially expressed transcripts, and putative functions could be assigned for 202 transcripts. A heat map of differentially expressed transcript profiles indicated the majority (78.5%) were upregulated in transmitters and/or acquirers, suggesting that the acquisition of MFSV induced transcript expression in *G. nigrifrons* (Fig. 1). For a subset of the differentially expressed transcripts (n = 57), the response was opposite between at least two transmission groups.

### MFSV-infected *G. nigrifrons* show elevated transcription of cytoskeleton organization and immunity genes

Of the 891 *G. nigrifrons* transcripts differentially expressed among transmission groups, 38% (n = 338) were upregulated in both acquirers and transmitters relative to non-acquirers and healthy control. In contrast, only 28 transcripts were downregulated in acquirers and transmitters. To examine whether the differentially regulated transcripts were associated with specific biological processes, functional annotation was assessed using the DAVID clustering tool [26]. Six significant annotation clusters were identified, including clusters containing transcripts that encode proteins involved in nucleotide binding, nuclear-transcribed mRNA metabolism, and with GTPase activity (Table 2). The nucleotide binding cluster was the most significant, and contained transcripts with a variety of putative functions without an obvious interconnection. A cellular stress response cluster was also identified, suggesting that a basal response is elicited by the insect host to intracellular virus infection.

**Table 2:** DAVID functional annotation clusters of *G. nigrifrons* transcripts upregulated in transmitters and acquirers relative to non-acquirers and the healthy control.

Annotation Cluster (representative annotation terms)	Transcript count^a^	Enrichment score^b^
1 Nucleotide binding	33	4.93
2 ATPase, AAA+ cluster	20	1.76
3 Cytoskeleton organization	19	1.43
4 Cellular stress response	15	1.8
5 Nuclear-transcribed mRNA metabolism	8	2.1
6 GTPase activity	8	1.43

a Number of transcripts in each significant annotation cluster.

b DAVID enrichment score for each significant annotation cluster.

Notable was a cluster of 19 transcripts functioning in cytoskeleton organization. Virus-induced cytoskeleton reorganization is a common strategy for virus transport to replication sites in insect cells [37]–[41]. For example, the release of *Rice gall dwarf virus* from cultured insect vector cells requires virus association with microtubules [42]. In addition, tubular structures induced by the *Rice dwarf virus* Pns10 protein facilitate virus replication in the vector [43]. Little information is currently available on the intracellular movement of plant-infecting rhabdoviruses in their insect hosts. We speculate that MFSV associates with leafhopper cytoskeleton components to allow movement of the virus into intracellular destinations of the host for replication.

An ATPase, AAA+ cluster was also upregulated in acquirers and transmitters. Based on the *Drosopohila* orthologs in this cluster, it contains transcripts that encode proteins belonging to several immune-related families [44]–[46]. These include a caspase (FBtr0085482), a clip-domain serine protease (FBtr0080000), a multidrug-resistance protein (FBtr0090025) and Dicer-2 of the small RNA regulatory pathway (FBtr0086904). Immune-related transcripts not in the ATPase, AAA+ cluster were also identified, including a MD2-like receptor (FBtr0085723) and a member of the Toll pathway (FBtr0075607). Other studies indicate a significant component of the insect host transcriptome response to persistent propagatively transmitted viruses is immune-related [11], [18], [47]. Moreover, the induction of immune transcripts was a major response of *G. nigrifrons* fed on MFSV-infected maize for shorter durations of 4 h and 1 wk, although the response involved a different set of immune transcripts [19].

Four transcripts encoding proteins with significant homology to viral A-type inclusion proteins (ATIPs) were upregulated in the MFSV-infected leafhoppers (i.e., transmitters and acquirers). Three of the four transcripts had a best match to different regions of a *Trichomonas vaginalis* ATIP nucleotide sequence (NCBI protein accession XP_001319570) and one best matched a *Clostridium botulinum* ATIP (NCBI protein accession YP_001390559.1). Inclusions are of interest since they are used by viruses to concentrate cellular and viral proteins, thereby representing sites of virus replication and assembly [48], [49]. Plant-infecting rhabdoviruses, particularly nucleorhabdoviruses, are known to form electron-dense inclusions inside the nucleus of infected cells, known as viroplasms [4]. Some plant RNA viruses induce the rearrangement of host membranes structures to form the viroplasm [48], which is consistent with the upregulation of cytoskeleton reorganization transcripts in acquirers and transmitters. Little is understood about the mechanism of viroplasm formation; however, in most cases it involves the recruitment of both virus components and host machinery [48], [50]. While there were no ATIP sequence signatures in the MFSV genome, it is conceivable that MFSV recruits host leafhopper ATIPS to inclusion sites. The putative *G. nigrifrons* ATIPs did not show significant nucleotide similarity to other insect species in the nr database; however, a putative ATIP was abundantly found in the midgut of the planthopper, *Nilaparvata lugens* [51], suggesting they may be pervasive in hemipteran insects of the suborder Auchenorrhyncha. Future targeted studies will be needed to disentangle the function roles (if any) of these putative ATIPs in virus replication and assembly.

### Similar transcript expression in *G. nigrifrons* acquirers or transmitters

Although MFSV replicates in both *G. nigrifrons* acquirers and transmitters, only the transmitters were empirically found to transmit the virus to new host plants [10]. Compared to the healthy control, more transcripts were upregulated than downregulated in acquirers (175 and 27, respectively) and transmitters (135 and 20, respectively). For the acquirers, no DAVID annotation clusters were identified and only 25 upregulated and 2 downregulated transcripts could be assigned a putative function using BLASTx (*E*-value < 10^−10^). Of interest were two induced dolichyldiphosphatases. These enzymes participate in N-glycan biosynthesis, which plays a critical role in cell recognition and adhesion, with carbohydrate-dependent interactions being essential for immune function [52].

Two significant DAVID annotation clusters were among the transcripts upregulated in transmitters only: one for cellular component movement (8 transcripts; score = 1.38) and one for intracellular signaling (8 transcripts; score = 1.62). MFSV appears to take a neurotropic route from the midgut into the salivary glands in their leafhopper vectors, similar to that of some vertebrate-infecting rhabdoviruses [1], [33]. Transcripts in these clusters may be important for facilitating this virus movement into the salivary glands.

Few genes have been directly implicated in insect host transmission efficiency of rhabdoviruses. Most of the available information is for the *Sigma viruses*, a clade of viruses in the family *Rhabdoviridae*, which naturally infects dipterans. In *D. melanogaster*, resistance alleles in the genes *ref(2)p* and *CHKov* 1 and 2 explain a large amount of the genetic variation in *Sigma virus* susceptibility [53]–[55]. However, orthologs of each of these genes were not detected in the *G. nigrifrons* transcriptome [11], [19] nor were transcripts of similar function differentially regulated between acquirers and transmitters.

Unlike non-acquirers, which can be easily distinguished from acquirers and transmitters on multiple levels, the distinction between acquirers and transmitters is based only on transmission efficacy and may not correlate perfectly with genotype. It is possible that a substantial proportion of the acquirers are capable vectors, but did not transmit MFSV to test plants during the one week inoculation period. Inability to accurately detect transmitters would skew the resultant expression values for the acquirers. The relatively small number of transcripts differentially expressed between acquirers and transmitters lend support this possibility. Moreover, the differences between the two transmission groups are likely due to one or a few genetic differences, and the gene(s) of interest may not be obvious using the transcriptome approach. Further studies are needed to identify factors that potentially confound our phenotype-driven transmission categories and influence the genetic analyses. For instance, RNAi knockdown of transcripts upregulated only the transmitters may implicate a gene in *G. nigrifrons* vector competence if the overall rate of MFSV transmission declines significantly.

### Sequence divergence among *G. nigrifrons* transmission groups is not linked with differential expression

**Figure 2:**
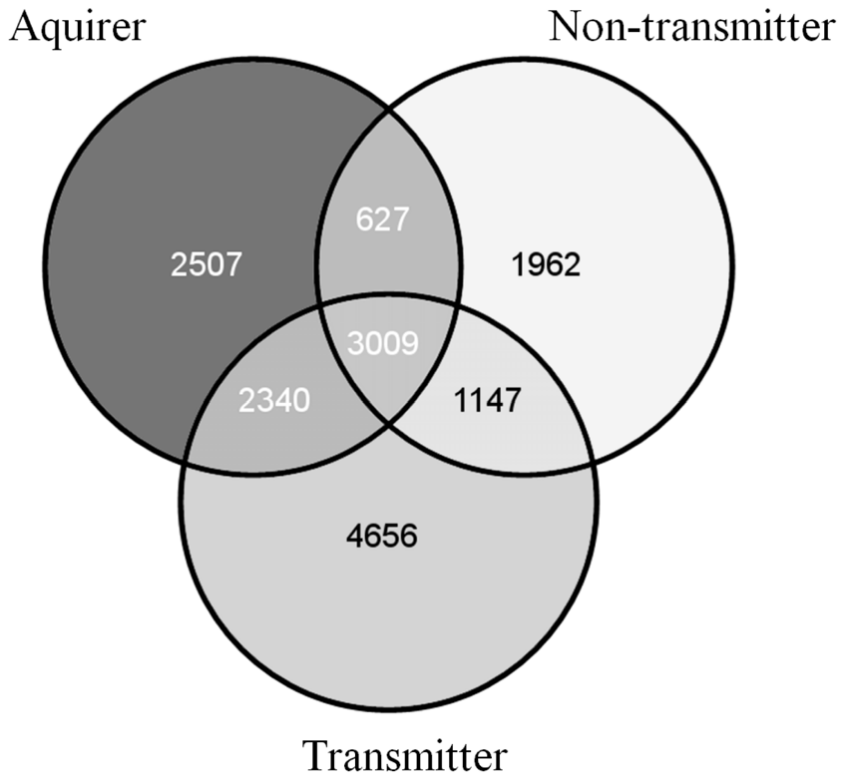
Venn diagram showing the partitioning of the 16,248 fixed homozygous polymorphisms detected among *G. nigrifrons* transmitters, acquirers, and non-acquirers.

To determine if the differentially expressed *G. nigrifrons* transcripts had more nucleotide polymorphisms than non-differentially expressed ones, we assayed sequences divergence between the transcriptomes of transmitters, acquirers and non-acquirers and the reference transcriptome, separately. In comparison to the *G. nigrifrons* reference transcriptome, 16,248 fixed homozygous polymorphisms were identified in 6,020 transcripts (Fig. 2). The transmitter transcriptome contained a greater number of nucleotide differences (n = 11,152) than acquirers (n = 8,483) and non-acquirers (n = 6,745). Among transmission groups, 150 differentially expressed transcripts (17%) contained at least one SNP. However, the sequence divergence in the differentially expressed transcripts was not significantly different than a uniform distribution [χ^2^ = 0.383 (1), *P* = 0. 536].

### MFSV transcript accumulation is similar between transmitters and acquirers

Virus titer may be an important factor governing transmission of rhabdoviruses [56]. It is conceivable that differences in MFSV transmission capability among leafhoppers reflected differences in virus transcript abundance. We searched for differences in transcript accumulation among the seven genes, which may provide insights into *G. nigrifrons* transmission capacity. As expected, MFSV was not detected in the non-acquirers and the healthy control. For both transmitters and acquirers, all seven transcripts were expressed but there was no interaction between transmission group and MFSV transcript abundance (*P*>0.05, for both RT-qPCR and RNA-Seq), indicating that the accumulation patterns were independent of the transmission group. It is still possible that differences in virus titer exist between the two transmission groups but could not be discerned by assaying only transcript abundance. Future studies are therefore needed to quantify the MFSV positive-sense RNA in purified virion.

### Expression of MFSV transcripts is not attenuated across the viral genome in *G. nigrifrons*

**Figure 3:**
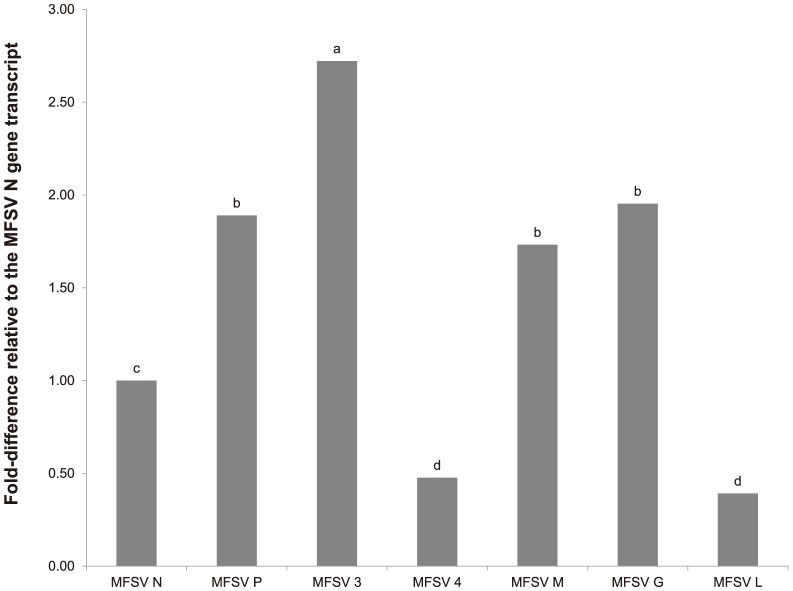
MFSV transcript accumulation in *G. nigrifrons* transmitters and acquirers. Accumulation of MFSV transcripts relative to the N gene was determined using RT-qPCR. Transcript accumulation was calculated according to the 2^−ΔΔCt^ algorithm using the *ribosomal protein S13* (RPS13) gene expression as the calibrator. Means for relative accumulation of each gene are shown. Bars with different letters were significantly different using LSD (*P*<0.05).

The MFSV genome encodes seven distinct genes in the order 3′-N-P-3-4-M-G-L-5′. Five genes are structural (N, P, M, G, and L) and two are non-structural (3 and 4) [20]. In animal rhabdoviruses, the expression of structural genes is primarily regulated by gene order, with decreasing levels of viral transcripts for genes from the 3′ end to 5′ end of the genome [57], [58]. The MFSV 4 and L transcripts accumulated at similar levels and were present at significantly lower levels than MFSV N transcript in the insect host (*P*<0.01) (Fig. 3). In contrast, the MFSV P, 3, M, and G transcripts accumulated to higher levels than the MFSV N transcript (*P*<0.01), with accumulation of MFSV 3 significantly higher than P, M, and G. These results indicate that MFSV gene transcription is not attenuated across the viral genome in the infected insect host, and suggests that the virus uses a different means to regulate transcript expression.

The greater accumulation of MFSV P, M, and G transcripts relative to the N transcript suggests their encoded proteins have active roles in addition to their structural roles in the leafhopper host. The M proteins of animal-infecting rhabdoviruses have been implicated in modulation of host cell transcription [59], [60]. Transcripts corresponding to the *Rabies virus* G gene are associated with the cellular poly(rC)-binding protein 2 (PCBP2), a multi-functional RNA-binding protein that regulates mRNA stability and translation [61]. The aforementioned DAVID analyses identified an enriched cluster of transcripts involved in nuclear-transcribed mRNA metabolism (8 transcripts; score = 2.1) as upregulated in both *G. nigrifrons* acquirers and transmitters. The P proteins of animal-infecting rhabdoviruses inhibit the immune response of host cells, particularly the IFN/STAT signaling pathway [62]. While we did not detect altered expression of INF/STAT pathway transcripts in transmitters or acquirers relative to non-acquirers or negative control leafhoppers, several innate immune-related transcripts were highly expressed in *G. nigrifrons* in response to MFSV infection.

The functions of the non-structural MFSV 3 and 4 genes remains to be elucidated [20], but we expected at least one of the genes to be important for virus movement in the plant [63]. The reduced accumulation of the MFSV 4 relative to the N transcripts in *G. nigrifrons* suggests that its role might be more important in the plant than in the insect host. Non-structural genes located between the P and M genes have been identified in many plant-infecting rhabdoviruses but not in animal-infecting rhabdoviruses [64]. It has been speculated that genes located in this portion of the genome play roles unique to the plant segment of the virus life [4], [63]. Interestingly, the protein encoded by the MFSV 4 has a molecular mass of 37.2 kDa [20], similar to the movement protein (MP) of SYNV sc4 [65], [66]. In addition, based on secondary structure predictions, the MFSV 4 protein shares similarity to the consensus core structure of 30K superfamily of viral MPs [63], [67]. For the MFSV 3 gene, its high expression relative to the N gene suggests that it may play an important role in MFSV infection of the insect host. Future studies are needed to disentangle the functional role of the MFSV 3 gene in virus infection and transmission.

### MFSV sequence divergence between *G. nigrifrons* transmission groups

The capability of *G. nigrifrons* to transmit MFSV may reflect differences in the virus rather than the insect host. Within infected hosts, an RNA virus exists as a population of closely related genotypes that arise through errors during genome replication that generate genetic variants [68], [69]. Genome sequence polymorphisms can affect viral pathogenesis, virulence, and transmission, and have been identified as one of the major forces driving adaption of RNA viruses to new hosts [70]–[72]. We identified 22 fixed SNPs among acquirers, transmitters, and the reference 13,782 bp MFSV genome from virus isolated from maize [20]. SNPs were identified in all genes except MFSV 3 (Table 3). Only two of the SNP produced nonsynonymous changes in the viral sequence relative to the reference genome: a glutamic acid to aspartic acid change at residue 461 of the MFSV N protein; and a methionine to threonine change at residue 233 of the MFSV L protein. No fixed nucleotide differences were identified between acquirers and transmitters. The MFSV reference genome sequence was determined from MFSV isolated from infected plant tissue, and may reflect a viral genome sequence that is not capable of infecting in leafhoppers, and the identified SNP may be advantageous for MFSV infection of leafhoppers. No fixed SNPs were identified between acquirers and transmitters, although seven were detected at >80% frequency (Table 2). It is possible that these SNPs represent fixed differences, with the lower frequencies attributed to the difficulties in discerning among individual transmitters from acquirers.

**Table 3:** MFSV sequence divergence between *G. nigrifrons* transmission groups^a^.

MFSV Gene	Nucleotide change				Freq (%)	Trans. Group
	Ref^a^	SNP variant^b^	Genome position^c^	coding region		
N^d^	T	C	685	Y	100	T,A
	T	C	700	Y	100	T,A
	A	C	1636	Y^i^	99.8	T,A
	A	T	1677	Y	99.7	T,A
P^e^	T	C	2140	Y	87.2	T
	A	G	2770	Y	99.7	T,A
	A	G	2836	Y	87.2	T
4	T	C	4221	Y	99.9	T,A
	C	T	4377	Y	98.3	T,A
	G	A	4525	N	99.9	T,A
	A	G	3922	Y	88.5	T
M^f^	C	T	4794	Y	99.4	T,A
	A	G	5175	Y	100	T,A
G^g^	A	G	6290	Y	99.8	T,A
	T	C	7286	Y	88.0	T,A
	T	C	7508	Y	87.6	T,A
	T	C	7555	N	92.8	T,A
	T	C	7556	N	92.8	T,A
	T	C	7561	N	86.8	T,A
	T	C	7565	N	87.6	T,A
	T	C	7587	N	87.7	T,A
	T	C	7590	N	87.4	T,A
L^h^	T	C	8361	Y^j^	100	T,A
	C	T	8645	Y	99.7	T,A
	A	C	9498	Y	99.8	T,A
	G	A	10030	Y	85.8	T
	A	C	10594	Y	100	T,A
	C	T	12032	Y	99.5	T,A
	T	C	13215	Y	99.7	T,A
	T	C	13525	N	100	T,A
	T	C	13528	N	100	T,A
	T	C	13548	N	100	T,A
	T	C	13595	N	82.3	T
	T	C	13597	N	80.9	T
	T	C	13600	N	82.4	T

a Nucleotide composition of the reference MFSV transcriptome [20].

b Single nucleotide polymorphism of the acquirer/transmitter relative to the MFSV reference sequence.

c Nucleotide position of the single nucleotide polymorphism in the MFSV reference sequence.

d *Nucleocapsid*.

e *Phospoprotein*.

f matrix.

g Glycoprotein.

h RNA *polymerase protein*.

i Results in Glutamate to Aspartate change at position 461 in the deduced amino acid sequence.

j Results in Methionine to Threonine change at position 233 in the deduced amino acid sequence.

Genome sequence polymorphisms (SNPs) were identified using the quality variant detection re-sequencing function in the CLC Bio. Specific nucleotide changes are indicated for each MFSV gene along with their position in the genome.

### Accession Numbers

The raw sequence reads can be retrieved from the NCBI short sequence read archive under the accession number SRP032742

## Conclusions

Genetic components in both the vector and virus and their interactions are major determinants of whether an individual within a species can transmit a virus. In this study, we used RNA-Seq to explore genetic changes in *G. nigrifrons* and MFSV that may be associated with the ability of the insect vector to acquire and transmit the virus. The consistency in MFSV transmission rates across replicates and studies provides evidence that intraspecific transmission capacity is a least partially genetically determined. Non-acquirers were distinguished from acquirers and transmitters, with substantial differences in the regulation of cytoskeleton organization and immunity genes. Genetic differences between acquirers and transmitters were more limited, which may be due to inherent difficulties discriminating between the two groups and/or the likelihood that the transmitter phenotype results from one or a few genetic differences. The competence of infected leafhoppers to transmit MFSV does not appear associated with virus transcript abundance in leafhoppers or sequence polymorphisms in the viral genome. However, the non-structural MFSV 3 gene was expressed at unexpectedly high levels in infected leafhoppers, suggesting it plays an active role in the infection of the insect host. Further studies are needed to identify factors that potentially confound our phenotype-driven transmission categories and influence the genetic analyses.

## Supporting Information Legends

Table S1 The primer pair sequences for targeted *G. nigrifrons* mRNAs designed using Primer3.
(XLSX)

Table S2 The 891 G. nigrifrons transcripts that had significantly different transcript accumulation among transmission groups. Each transcript was a minimum 2-RPKM up or down regulated relative to the healthy control.
(XLSX)
